# A Curious Mistake

**Published:** 1883-01

**Authors:** 


### A CURIOUS MISTAKE.

In our November (1882) issue we credited some "Lycopodium Clinical Cases" to Dr. Clarence Willard Butler, of Montclair, N. J. Now Dr. Butler writes us that he never wrote these interesting cases! Will the author kindly send us his name, and accept our apologies for the mistake?

